# GTSE1 is possibly involved in the DNA damage repair and cisplatin resistance in osteosarcoma

**DOI:** 10.1186/s13018-021-02859-8

**Published:** 2021-12-07

**Authors:** Chaofan Xie, Wei Xiang, Huiyong Shen, Jingnan Shen

**Affiliations:** 1 Department of Orthopaedic, The First Affiliated Hospital of Sun Yat-Sen University, Guangzhou, 510000 Guangdong People’s Republic of China; 2 Department of Orthopaedic, The Eighth Affiliated Hospital of Sun Yat-Sen University, No. 3025, Shennan Middle Road, Futian District, Shenzhen, 518033 Guangdong People’s Republic of China; 3 Department of Muscularskeletal Oncology, The First Affiliated Hospital of Sun Yat-Sen University, No. 58, Zhongshan 2nd Road, Guangzhou, 510000 Guangdong People’s Republic of China

**Keywords:** Osteosarcoma, GTSE1, DNA repair, Cisplatin, Drug resistance

## Abstract

**Background:**

G2 and S phase-expressed-1 (GTSE1) negatively regulates the tumor-suppressive protein p53 and is potentially correlated with chemoresistance of cancer cells. This study aims to explore the effect of GTSE1 on the DNA damage repair and cisplatin (CDDP) resistance in osteosarcoma (OS).

**Materials and methods:**

Expression of GTSE1 in OS was predicted in bioinformatics system GEPIA and then validated in clinically obtained tissues and acquired cell lines using RT-qPCR, immunohistochemical staining, and western blot assays. Gain- and loss-of-function studies of GTSE1 were performed in MG-63 and 143B cells to examine its function in cell cycle progression, DNA replication, and CDDP resistance. Stably transfected MG-63 cells were administrated into mice, followed by CDDP treatment to detect the role of GTSE1 in CDDP resistance in vivo.

**Results:**

GTSE1 was highly expressed in patients with OS and correlated with poor survival according to the bioinformatics predictions. Elevated GTSE1 expression was detected in OS tissues and cell lines. GTSE1 silencing reduced S/G2 transition and DNA replication, and it increased the CDDP sensitivity and decreased the expression of DNA repair-related biomarkers in MG-63 cells. GTSE1 overexpression in 143B cells led to inverse trends. In vivo, downregulation of GTSE1 strengthened the treating effect of CDDP and significantly repressed growth of xenograft tumors in nude mice. However, overexpression of GTSE1 blocked the anti-tumor effect of CDDP.

**Conclusion:**

This study demonstrates that GTSE1 is possibly involved in the DNA damage repair and cisplatin resistance in OS.

## Background

Osteosarcoma (OS), characterized by high recurrence and metastasis rates, is the most common primary bone tumor that predominately affects children, adolescents, and young adults, and advanced patients with metastasis share an unfavorable prognosis [1]. OS is likely to occur in the long bones, mainly including distal femur (43%), proximal tibia (23%), and humerus (10%), which are sites providing the most proliferative plates [2, 3]. Within these long bones, the tumor is commonly (90%) located in the metaphysis and appears as a palpable mass which is related with severe pain that is intense enough to wake patients from sleep [4, 5].

The current conventional treatments, including surgical resection and adjuvant chemotherapy, have improved the survival probability of patients, but the 5-year survival rate of patients with metastatic and recurrent disease remains unsatisfactory [5, 6]. Introduction of chemotherapy regimens such as cisplatin (CDDP), methotrexate, and doxorubicin significantly elevated prognosis for patients with localized OS [5, 7]. However, the survival rate has not improved during the past 2 decades [8], mainly owing to the acquired resistance of patients to chemotherapy [9, 10]. The chemotherapies induce DNA damages either directly (CDDP) or indirectly (doxorubicin) [11]. Resistance mechanisms of DNA damage can lead to OS chemotherapy unresponsiveness, and therefore, overcoming DNA damage repair may enhance the sensitivity of tumor cells to chemo-drugs and enhance the treating outcome.

G2 and S phase-expressed-1 (GTSE1) is a p53-inducible gene located in chromosome 22q13.2-q13.3 and is specifically expressed in the S and G2 phases of the cell cycle [12, 13]. GTSE1 co-localizes with tubulin or microtubules and regulates microtubule dynamics by suppressing the microtubule depolymerase MCAK, which is fundamental for chromosome stability and alignment and spindle integrity during mitosis [14]. Besides, GTSE1 can also negatively regulate nuclear accumulation and promote cytoplasmic localization and degradation of p53 owing to its nucleocytoplasmic shuttling ability [12, 15]. GTSE1 accumulates in the nucleus in response to DNA damage and downregulates p53, which allows cells to avoid apoptosis and promotes the G2/M transition, resulting in tumor development [16, 17]. GTSE1 has been reported to confer CDDP resistance to gastric cancer cells through inhibiting the p53 signaling [18]. Increased expression of GTSE1 was found in several human malignancies, such as lung cancer [19], breast carcinoma [20], and hepatocellular carcinoma [21] and was correlated with unfavorable prognosis. However, the GTSE1 expression in OS and its link with drug resistance remain unexplored. This study intended to explore the function of GTSE1 in DNA damage and CDDP resistance in OS.

## Materials and methods

### Clinical samples

OS tissues and the paired adjacent normal tissues were collected from 15 patients treated at the First Affiliated Hospital of Sun Yat-Sen University from November 2018 to May 2020. There were 9 males and 6 females, all of which were diagnosed as OS for the first time and without a history of radio- or chemo-therapy. The tissue samples were collected during surgery, immediately frozen in liquid nitrogen, and stored at − 80 °C until further use. This research was performed with the approval of Ethics Committee of the First Affiliated Hospital of Sun Yat-Sen University and in compliance with the *Declaration of Helsinki.* Each eligible participant signed the informed consent.

### Cell culture

An osteoblast cell line hFOB 1.19 (CRL-11372) and four OS cell lines MNNG (CRL-1547), MG-63 (CRL-1427), 143B (CRL-8303), and SaOS-2 (HTB-85) were acquired from American Type Culture Collection (Manassas, VA, USA). All cells were cultured in Dulbecco’s modified Eagle’s medium (DMEM) containing 10% fetal bovine serum (FBS), 100 U/mL penicillin, and 100 μg/mL streptomycin in a humidified incubator at 37 °C with 5% CO_2_. The medium was renewed every 2 days.

### Cell transfection

Short hairpin (sh) RNA and overexpression vector of GTSE1 (sh-GTSE1; oe-GTSE1), and the negative control (NC) vectors (sh-NC and oe-NC) were all procured from RiboBio Co., Ltd. (Guangzhou, Guangdong China). MG-63 cells were transfected with sh-GTSE1 or sh-NC, and 143B cells were transfected with oe-NC or oe-GTSE1 for in vitro experiments. MG-63 cells transfected with oe-GTSE1, sh-GTSE1, and empty vector (EV) were used for in vivo experiments. All transfections were conducted using the lipofectamine 2000 kit (Thermo Fisher Scientific Inc., Waltham, MA, USA). Stably transfected cells were screened using 2 μg/mL puromycin (Sigma-Aldrich, St Louis, MO, USA). Successfully transfected cells were cultured in DMEM at 37 °C with 5% CO_2_ for subsequent use.

### Reverse transcription quantitative polymerase chain reaction (RT-qPCR)

Total RNA from OS and normal tissues or cells was isolated by the TRIzol reagent (Thermo Fisher Scientific). Complementary DNA (cDNA) was synthesized using a Primescript RT kit (Takara Holdings Inc., Kyoto, Japan). Expression of GTSE1, EXO1, PLK4, and RFC4 mRNA was detected using the FastStart PCR master mix (Roche Ltd., Basel, Switzerland). The ABI Prism 7500 System (Life Technologies, Gaithersburg, MD, USA) was used for data collection and quantification analysis. Glyceraldehyde-3-phosphate dehydrogenase (GAPDH) was used as the endogenous loading. The primer sequences are listed in Table 1.

**Table 1:** Primer sequences for RT-qPCR

Primers	Sequence (5ʹ-3ʹ)
GTSE1	F: CTCTACCAGCAATCTCGCAAGG
	R: GACTTGCTGATGTTTGACAGAGG
EXO1	F: TCGGATCTCCTAGCTTTTGGCTG
	R: AGCTGTCTGCACATTCCTAGCC
PLK4	F: GACACCTCAGACTGAAACCGTAC
	R: GTCCTTCTGCAAATCTGGATGGC
RFC4	F: GGCAGCTTTAAGACGTACCATGG
	R: TCTGACAGAGGCTTGAAGCGGA
GAPDH	F: GTCTCCTCTGACTTCAACAGCG
	R: ACCACCCTGTTGCTGTAGCCAA

RT-qPCR, reverse transcription-quantitative polymerase chain reaction; GTSE1, G2 and S phase-expressed-1; EXO1, exonuclease 1; PLK4, polo-like kinase 4; RFC4, replication factor C subunit 4; GAPDH, glyceraldehyde-3-phosphate dehydrogenase

### Western blot analysis

Cells (1 × 10^6^) were lysed in RIPA lysis buffer (Solarbio Science & Technology Co., Ltd., Beijing, China) to collect total protein, and the protein concentration was examined using a bicinchoninic acid kit (Keygen Biotech Co., Ltd., Nanjing, Jiangsu, China) according to the instructions. An equal amount (60 μg) of protein sample was run on 12% SDS-PAGE and transferred onto PVDF membranes (Millipore, Billerica, MA, USA). After being blocked in 5% not-fat milk for 1 h, the membranes were hybridized with the primary antibodies GAPDH (1: 10,000, ab181602, Abcam), GTSE1 (1:1000, GTX66223, GeneTex, CA, USA), Cyclin D1 (1:1000, #2978S, Cell Signaling Technology (CST), Beverly, MA, USA), Cyclin E1 (1:1000, #20808S, CST), proliferating cell nuclear antigen (PCNA; 1:1000, #13110S), cleaved caspase 3 (1:500, ab32042), Bax (1:1000, GTX109683, GeneTex), γH2AX (1:5000, ab81299), and DNA-PKcs (1:1000, ab32566) at 4 °C overnight, and then with HRP-conjugated secondary antibody (1:5000, ab205718, Abcam Inc., Cambridge, MA, USA) at room temperature for 1 h. The protein bands were developed using the ECL reagent (Pierce, Rockford, IL, USA).

### Cell cycle analysis

After transfection, the cells (1 × 10^6^) were washed with phosphate-buffered saline (PBS) and fixed with 70% ethanol at 4 °C overnight. All samples were treated with 5 μL RNase A and 450 μL propidium iodide (PI, Keygen) in the dark at 22 °C for 1 h. The cell cycle distribution was analyzed by a flow cytometer (Applied Biosystems, Inc., Carlsbad, CA, USA).

### 5-Ethynyl-2ʹ-deoxyuridine (EdU) labeling assay

Proliferation activity of the cells was examined using an EdU labeling kit (RiboBio Co., Ltd., Guangzhou, Guangdong, China). In brief, MG-63 and 143B cells were seeded in 24-well plates (5 × 10^5^ cells per well) for 24 h and incubated with 50 μM EdU for 2 h. Cells were washed in PBS, fixed with 4% paraformaldehyde (PFA) for 30 min, penetrated in 0.5% Triton X-100 for 10 min, treated with 200 μL 1 × Apollo reaction mixture in the dark for 30 min, and finally treated with 200 μL 1 × Hoechst 33,342 for 30 min. The staining was observed under a fluorescence microscope (Olympus Optical Co., Ltd, Tokyo, Japan). Positively stained cells were counted under 5 random fields of views.

### Flow cytometry for cell apoptosis

Apoptosis of cells was examined using an Annexin V-fluorescein isothiocyanate (FITC)/PI kit (Invitrogen, Thermo Fisher Scientific). In short, the OS cells (1 × 10^6^) were washed with cold PBS and resuspended in 400 μL Annexin V-FITC binding buffer, and then stained with 5 μL Annexin V-FITC and 10 μL PI at 22 °C in the dark for 15 min. The FITC and PI fluorescence was examined using the flow cytometer. The apoptosis of cells was examined using the CellQuest software (BD Biosciences).

### Cell activity examination

MG-63 cells were allocated into Control group, sh-NC group, and sh-GTSE1 group, and 143B cells were allocated into oe-NC and oe-GTSE1 groups after corresponding transfections. The cells were seeded on 96-well plates at 5000 cells per well. After cell adherence, cells were treated with CDDP (Qilu Pharmaceutical Co., Ltd., Shandong) at different doses (0, 1, 2, 3, 4, and 5 μg/mL). After 24 h, each well was loaded with 10 μL cell counting kit-8 (CCK-8) reagent (BestBio, Shanghai, China) for 2 h of incubation at 37 °C. The optical density (OD) at 450 nm was determined using a microplate reader (EMax Plus, Molecular Devices, CA, USA). The 50% inhibitory concentration (IC_50_) was examined using the GraphPad Prism 5 software.

### Xenograft tumors in nude mice

MG-63 cells transfected with sh-GTSE1, oe-GTSE1, or EV were used for in vivo experiments. Twenty-four female BALB/c nude mice (3–4 weeks old, 15.5 ± 2.4 g) were procured from Laboratory Animal Center of Chinese Academy of Sciences (Shanghai, China). The animals were housed in specific-pathogen-free grade animal rooms in a 12-h dark/light cycle with free access to food and drinking water. After one week of acclimation, the mice were allocated into four groups (EV + PBS group, EV + CDDP group, sh-GTSE1 + CDDP group, and oe-GTSE1 + CDDP group), 6 in each. In the EV + PBS group, mice were subcutaneously injected with MG-63 cells (1 × 10^6^) transfected with EV and intraperitoneally injected with PBS weekly. In the EV + CDDP group, mice were subcutaneously injected with MG-63 cells (1 × 10^6^) transfected with EV and intraperitoneally injected with CDDP (3 mg/kg) weekly. In the sh-GTSE1 + CDDP group, mice were subcutaneously injected with MG-63 cells (1 × 10^6^) transfected with sh-GTSE1 and intraperitoneally injected with CDDP (3 mg/kg) weekly. In the oe-GTSE1 + CDDP group, mice were subcutaneously injected with MG-63 cells (1 × 10^6^) transfected with oe-GTSE1 and intraperitoneally injected with CDDP (3 mg/kg) weekly. After cell transplantation, the volume (*V*) of xenograft tumors was examined weekly as follows: *V* = *a* × *b*^2^/2, in which “*a*” refers to the length and “*b*” refers to the width. After 4 weeks, the mice were euthanized with 1% pentobarbital sodium (150 mg/kg) to collect the tumor tissues for weighing and histological examination. Animal procedures were approved by the Animal Ethics Committee of the First Affiliated Hospital of Sun Yat-Sen University and performed with the Guide for the Care and Use of Laboratory Animals (NIH, Bethesda, Maryland, USA).

### Immunohistochemical (IHC) staining

Collected tissue samples were fixed with 4% PFA, embedded in paraffin, and cut into 4-μm slices. The slices were dewaxed in xylene and rehydrated in ethanol, treated with 3% H_2_O_2_ to block the activity of endogenous peroxidases, and treated with citrate buffer for antigen retrieval. After that, the slices were then treated with normal goat serum and incubated with antibodies against GTSE1 (1:500, ab272670, Abcam), Ki-67 (1:100, 14-5698-82, Thermo Fisher Scientific) overnight at 4 °C and treated with the goat anti-rabbit IgG (1:2000, ab205718, Abcam) at room temperature for 1 h. The slices were incubated with 40 μL HRP-labeled streptavidin working solution at 37 °C for 15 min. The staining was developed using DAB, and the slices were counter-stained with hematoxylin, rehydrated in alcohol, sealed with neutral balsam, and observed under the microscope. The number of GTSE1- or Ki-67-positive cells was counted under the microscope with five random fields included.

### Terminal deoxynucleotidyl transferase (TdT)-mediated dUTP nick end labeling (TUNEL)

The tumor tissues were cut into 4-μm slices and labeled according to the instructions of a TUNEL kit (Roche, Indianapolis, IN, USA). In brief, the slices were routinely dewaxed, rehydrated, and permeabilized. After that, the slices were added with 20 mg/L protease K solution for 15 min and 500 μL TUNEL reaction mixture for 1 h of reaction in a wet box in the dark. Next, the slices were added with 50 μL converter-POD for 30 min reaction in the wet box. Thereafter, 100 μL DAB was added for a 10-min incubation. The slices were counter-stained with hematoxylin and observed under the microscope (Nikon Instruments Inc., Tokyo, Japan). The apoptosis rate in tissues was determined as follows: rate = number of apoptotic tissues/total cells.

### Statistical analysis

For each experiment, at least three repetitions were performed. Statistical analysis was conducted by SPSS 20.0 (IBM Corp. Armonk, NY, USA) and GraphPad Prism 8.02 (GraphPad Software, CA). Data were exhibited as the mean ± standard deviation (SD). Differences were analyzed by the paired or unpaired *t* test (two groups), or by one- or two-way analysis of variance (ANOVA) followed by Tukey’s multiple comparisons test (over two groups). *p* < 0.05 was considered to show statistical analysis.

## Results

### GTSE1 is highly expressed in OS and possibly correlated with unfavorable outcome

**Fig. 1:**
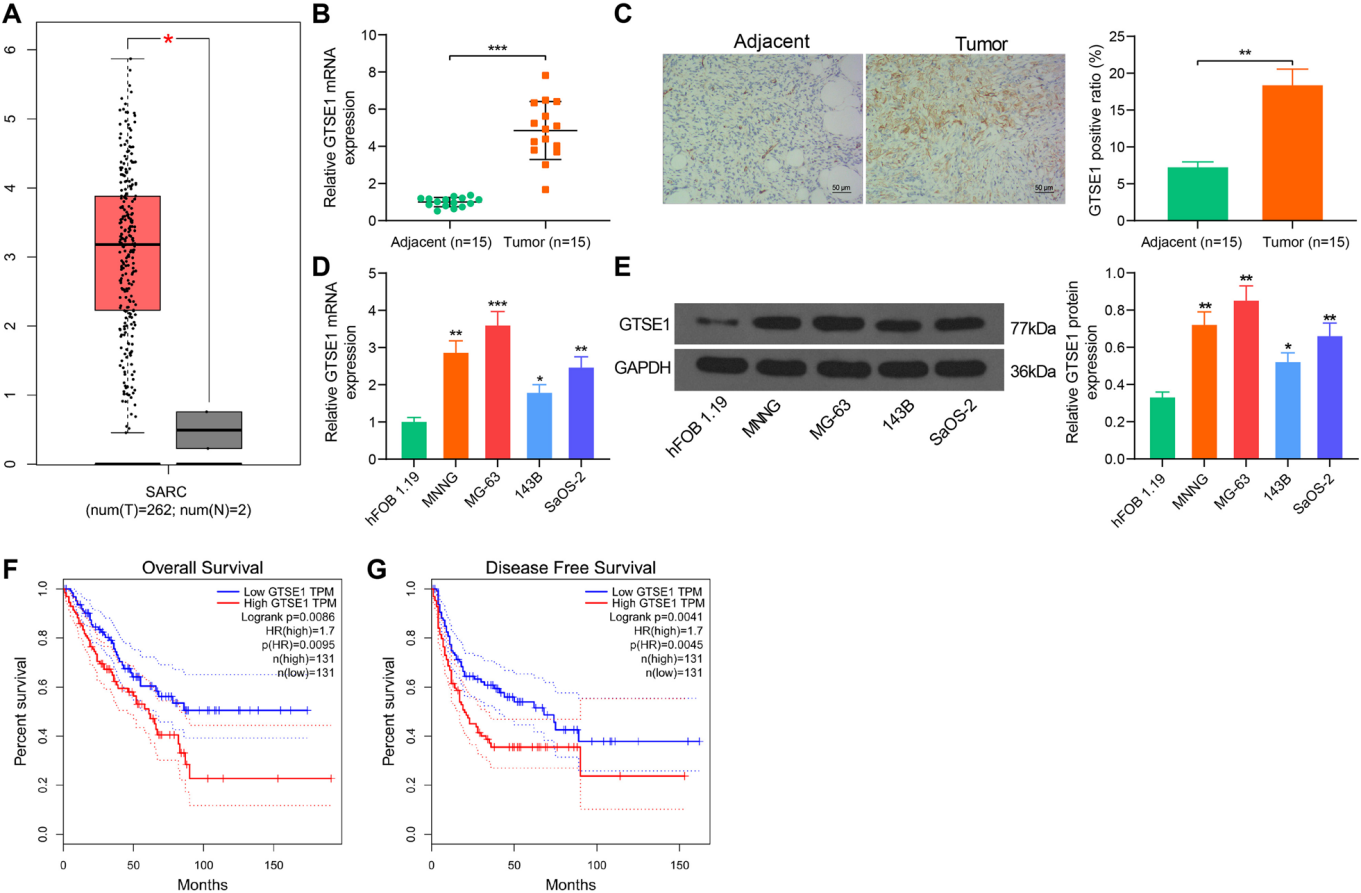
GTSE1 is highly expressed in OS and possibly correlates with unfavorable outcome. **A** expression profiling of GTSE1 in sarcoma in GEPIA; **B**, **C** expression of GTSE1 in OS tumor and para-tumorous tissues (*n* = 15) examined by RT-qPCR (**B**) and IHC staining (**C**), respectively (paired *t* test); D–E, expression of GTSE1 in OS cell lines (MNNG, MG-63, 143B, SaOS-2) and in normal hFOB 1.19 cells determined by RT-qPCR (**D**) and western blot analysis (**E**), respectively (one-way ANOVA vs. hFOB 1.19 cells); **F**, **G** overall survival (**F**) and disease-free survival (**G**) of patients with OS in the GEPIA database. Data were presented as the mean ± SD from three repetitions. *p < 0.05, **p < 0.01, ***p < 0.001

The expression profiling of GTSE1 in OS was first obtained in Gene Expression Profiling Interactive Analysis (GEPIA) (http://gepia.cancer-pku.cn/detail.php), a web-based tool to deliver fast and customizable functionalities based on tremendous amount of RNA sequencing data from large consortium projects such as the Cancer Genome Atlas (TCGA) and Genotype-Tissue Expression (GTEx) [22]. The GEPIA data suggested that GTSE1 was highly expressed in patients with sarcoma (Fig. 1A). In the clinical tissue samples, GTSE1 expression was elevated in OS tumor tissues relative to the adjacent tissues according to RT-qPCR and IHC staining assays (Fig. 1B, C). A similar trend was identified in the acquired cell lines where increased mRNA and protein expression of GTSE1 was detected in four OS cell lines compared to the normal hFOB 1.19 cells (Fig. 1D, E). In addition, data in GEPIA suggested that high expression of GTSE1 in patients with OS was correlated with reduced survival probability and increased risk of recurrence (Fig. 1F, G).

### GTSE1 promotes S/G2 transition and DNA replication in OS cells

**Fig. 2:**
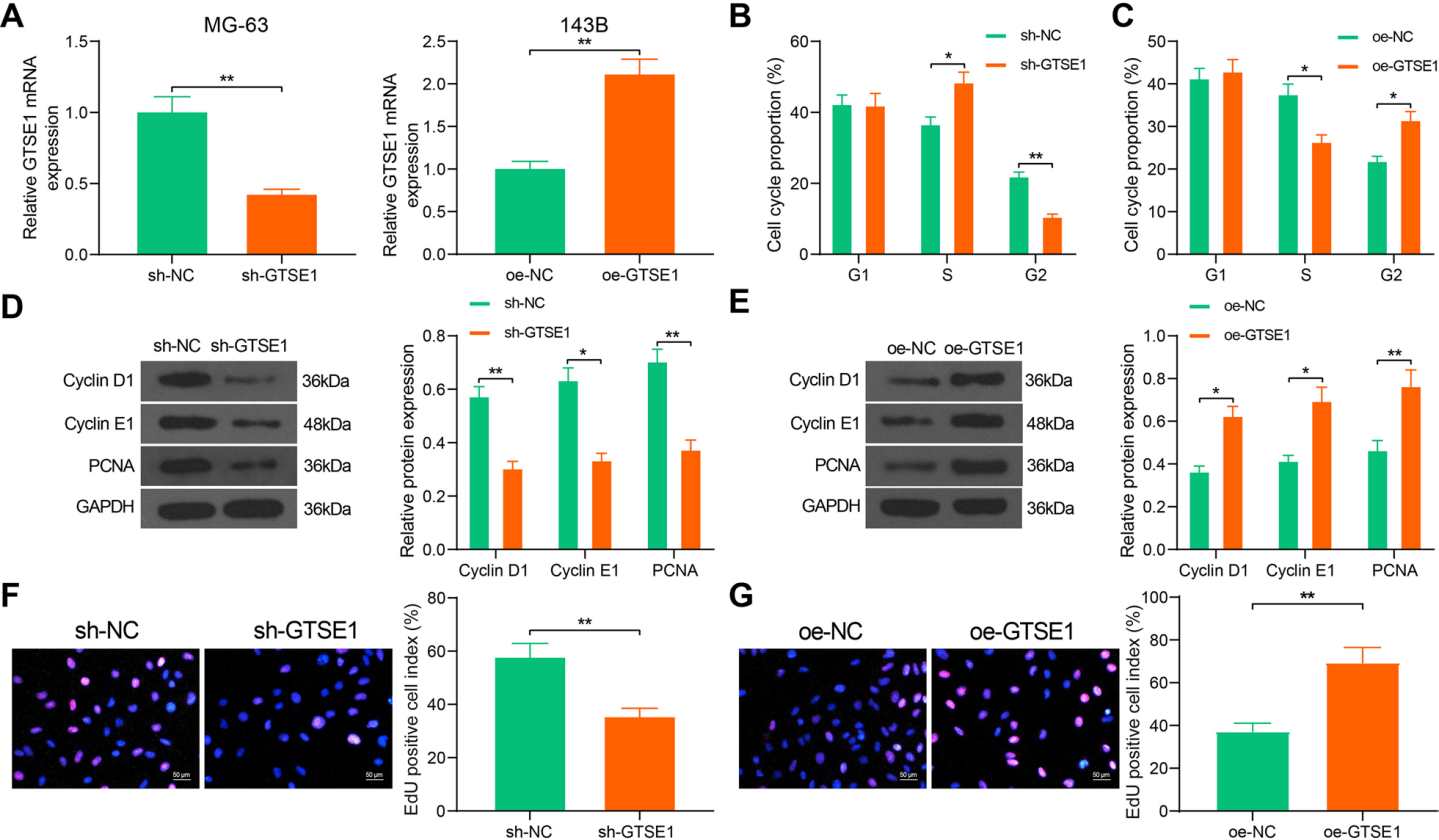
GTSE1 promotes S/G2 transition and DNA replication in OS cells. **A** mRNA expression of GTSE1 in MG-63 and 143B cells after sh-GTSE1 or oe-GTSE1 transfection, respectively, examined by RT-qPCR (unpaired *t* test); **B**, **C** cell cycle progression in MG-63 (**B**) and 143B (**C**) cells determined by flow cytometry (two-way ANOVA); D–E, protein levels of Cyclin D1, Cyclin E1, and PCNA in MG-63 (**D**) and 143B (**E**) cells determined by western blot analysis (two-way ANOVA); **F**, **G** DNA replication ability of MG-63 (**F**) and 143B (**G**) cells examined by EdU labeling assay (unpaired* t* test). Data were presented as the mean ± SD from three repetitions. *p < 0.05, **p < 0.01

According to the results in Fig. 1E, MG-63 cells with the highest GTSE1 expression were transfected with sh-GTSE1, whereas 143B cells with the lowest GTSE1 expression among the four OS cell lines were transfected with oe-GTSE1 for gain- and loss-of-function studies in vitro. The successful transfections were validated by RT-qPCR (Fig. 2A). Flow cytometry on cell cycle distribution suggested that knockdown of GTSE1 in MG-63 cells increased the number of cells at S phase but reduced the number of cells at G2 phase, indicating that GTSE1 silencing blocked the S/G2 transition in MG-63 cells (Fig. 2B). By contrast, overexpression of GTSE1 reduced the portion of 143B cells at S phase and increased the portion of cells at G2 phase (Fig. 2C). In the molecular perspective, downregulation of GTSE1 in MG-63 cells reduced the expression of S/G2 transition-related factors, including Cyclin D1 and Cyclin E1 and it also reduced the expression of DNA replication-related factor PCNA in cells (Fig. 2D). As expected, inverse results were found in 143B cells where GTSE1 was upregulated (Fig. 2E). The function of GTSE1 in DNA replication of cells was examined by the EdU labeling assay. It was indicated that silencing of GTSE1 reduced the number of EdU-positive (DNA replicating) MG-63 cells, whereas overexpression of GTSE1 increased the number EdU-positive 143B cells (Fig. 2F, G). These results indicated that GTSE1 is crucial for the S/G2 transition and DNA replication in OS cells.

### GTSE1 is correlated with CDDP sensitivity of OS cells

**Fig. 3:**
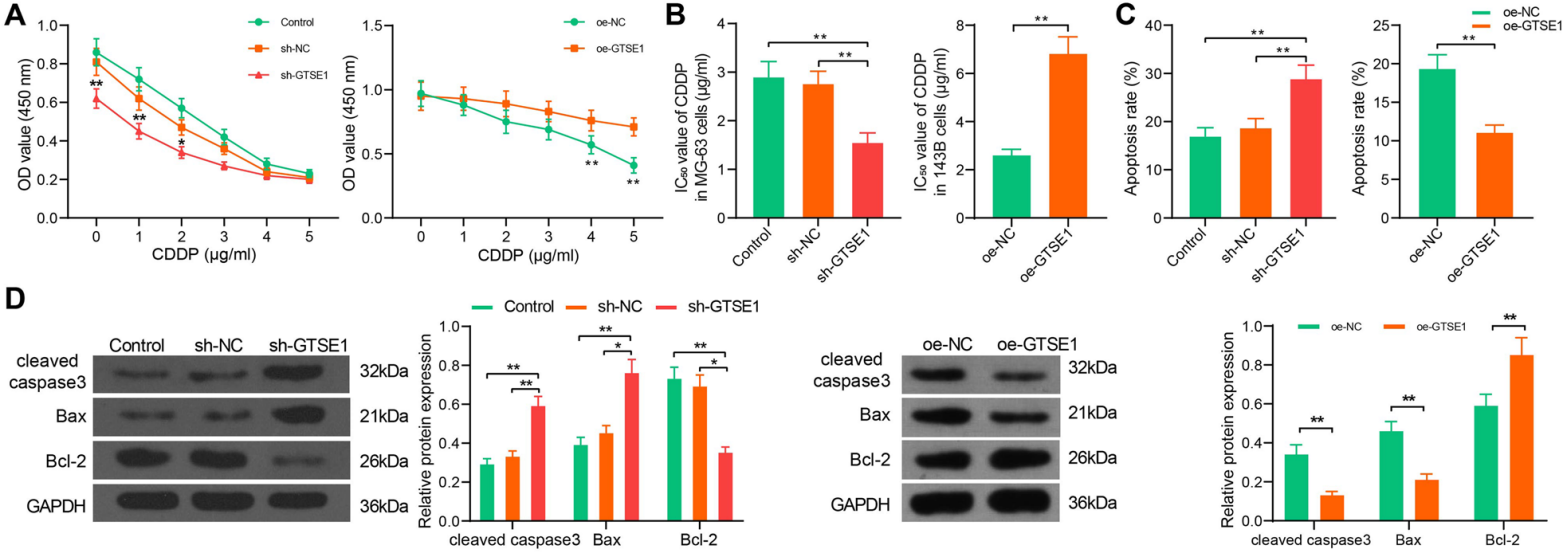
GTSE1 is correlated with CDDP sensitivity of OS cells. **A** Proliferation of MG-63 and 143B cells after different doses of CDDP treatment determined by the CCK-8 method (two-way ANOVA); **B** IC_50_ value of CDDP in each group of cells (two-way ANOVA); **C** apoptosis rate in each group of cells after CDDP treatment at 3 μg/mL determined by flow cytometry (one-way ANOVA); **D** protein levels of apoptosis-related factors cleaved caspase 3, Bax, and Bcl-2 in each group of cells quantified by western blot analysis (two-way ANOVA). Data were presented as the mean ± SD from three repetitions. *p < 0.05, **p < 0.01

To determine whether the GTSE1 expression is correlated with the CDDP resistance of OS cells, MG-63 cells transfected with sh-GTSE1 and sh-NC, and 143B cells transfected with oe-GTSE1 and oe-NC transfected were treated with different doses of CDDP. After 48 h, the IC_50_ value of CDDP was determined by the CCK-8 method. The IC_50_ value of CDDP in the Control group (without any transfection) was 2.89 μg/mL, and that in the sh-NC and sh-GTSE1 groups was 2.77 μg/mL and 1.54 μg/mL, respectively. By contrast, the IC_50_ value of CDDP in the oe-NC and oe-GTSE1 groups was 2.59 and 6.81, respectively (Fig. 3A, B). These results indicated that silencing of GTSE1 significantly reduced the IC_50_ value of CDDP, namely elevated the CDDP sensitivity of MG-63 cells, and overexpression of GTSE1 decreased the CDDP sensitivity of 143B cells. Next, cells in each group were treated with CDDP at 3 μg/mL for 48 h, and flow cytometry was performed to analysis cell apoptosis. Of note, GTSE1 downregulation significantly elevated cell apoptosis induced by CDDP. However, GTSE1 overexpression decreased apoptosis rate of 143B following CDDP treatment, indicating that GTSE1 increased the resistance of cells to CDDP (Fig. 3C). Western blot analysis on cell apoptosis-related factors indicated that silencing of GTSE1 significantly increased the expression of pro-apoptotic proteins cleaved caspase3 and Bax but reduced the expression of anti-apoptotic Bcl-2 in MG-63 cells. Still, overexpression of GTSE1 suppressed the levels of cleaved caspase3 and Bax but increased the level of Bcl-1 in 143B cells (Fig. 3D). These results, collectively, suggested that GTSE1 knockdown increases CDDP sensitivity in OS cells.

### GTSE1 enhances DNA damage repair-related biomarkers in OS cells and reduces CDDP-induced cell apoptosis

**Fig. 4:**
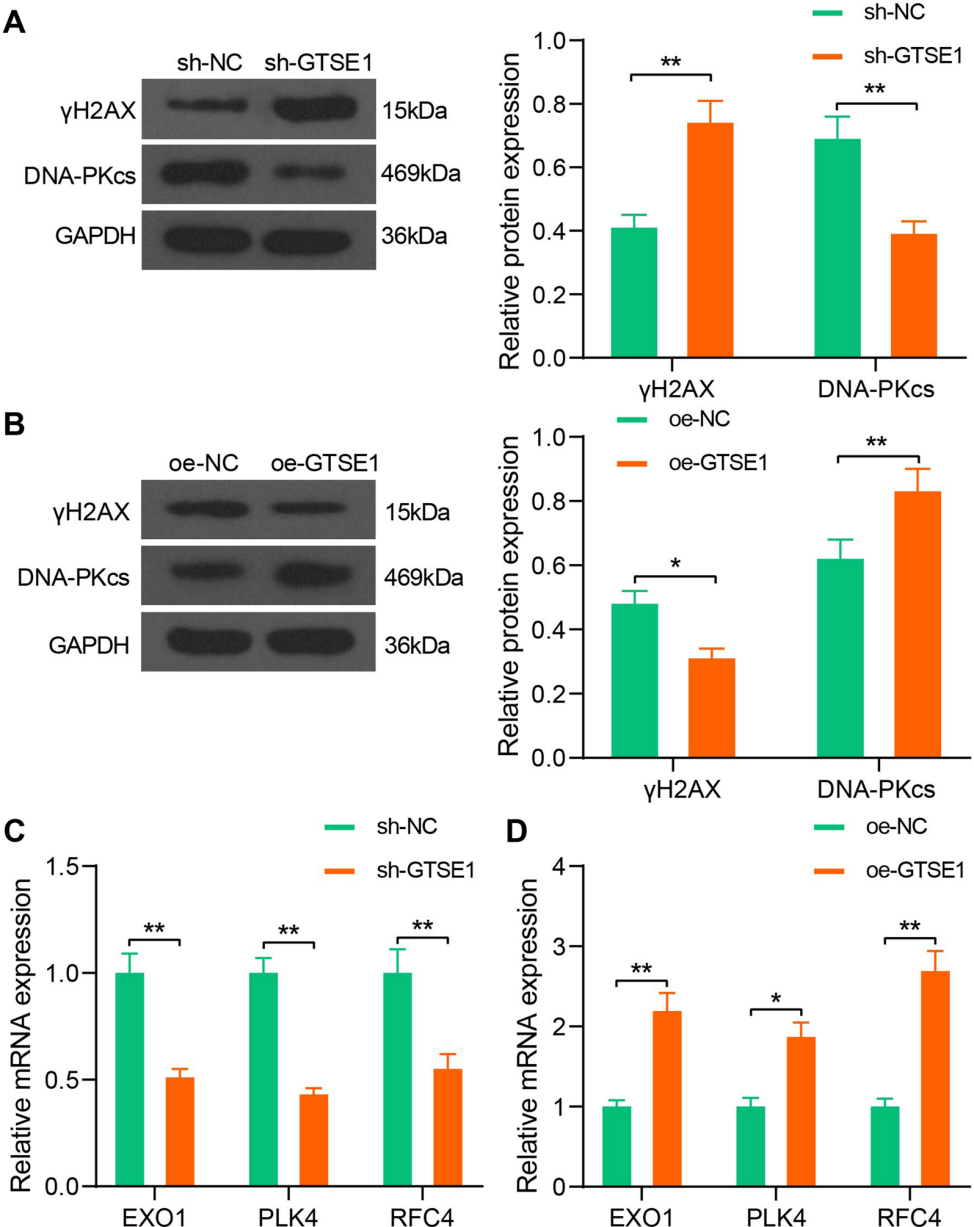
GTSE1 enhances DNA damage repair-related biomarker in OS cells and reduces CDDP-induced cell apoptosis. **A**, **B** Protein levels of γH2AX and DNA-PKcs in MG-63 (**A**) and 143B (**B**) cells determined by western blot analysis (two-way ANOVA); **C**, **D** mRNA expression of DNA repair-related factors EXO1, PLK4, and RFC4 in MG-63 (**C**) and 143B (**D**) cells determined by RT-qPCR (two-way ANOVA). Data were presented as the mean ± SD from three repetitions. *p < 0.05, **p < 0.01

The findings above indicated that GTSE1 is possibly necessary for DNA replication and CDDP resistance in OS cells. To validate this, MG-63 cells transfected with sh-GTSE1 and 143B cells transfected with oe-GTSE1 were treated with CDDP (3 μg/mL) to induce DNA damage. After that, the levels of γH2AX, a DNA double-strand break biomarker, and DNA-PKcs, a DNA damage repair biomarker, were determined using western blot analysis. After 48 h of CDDP treatment, the expression of γH2AX in MG-63 cells was significantly elevated, but the expression of DNA-PKcs was reduced (Fig. 4A). However, overexpression of GTSE1 led to an increase in DNA-PKcs while a decline in γH2AX in 143B cells (Fig. 4B), indicating that GTSE1 is possibly correlated with DNA damage repair in OS cells. Meanwhile, silencing of GTSE1 reduced the expression of DNA repair-related factors EXO1, PLK4, and RFC4 in MG-63 cells (Fig. 4C), and overexpression of GTSE1 led to inverse trends in 143B cells again (Fig. 4D).

### GTSE1 affects tumorigenesis of OS cells in nude mice following CDDP treatment

**Fig. 5:**
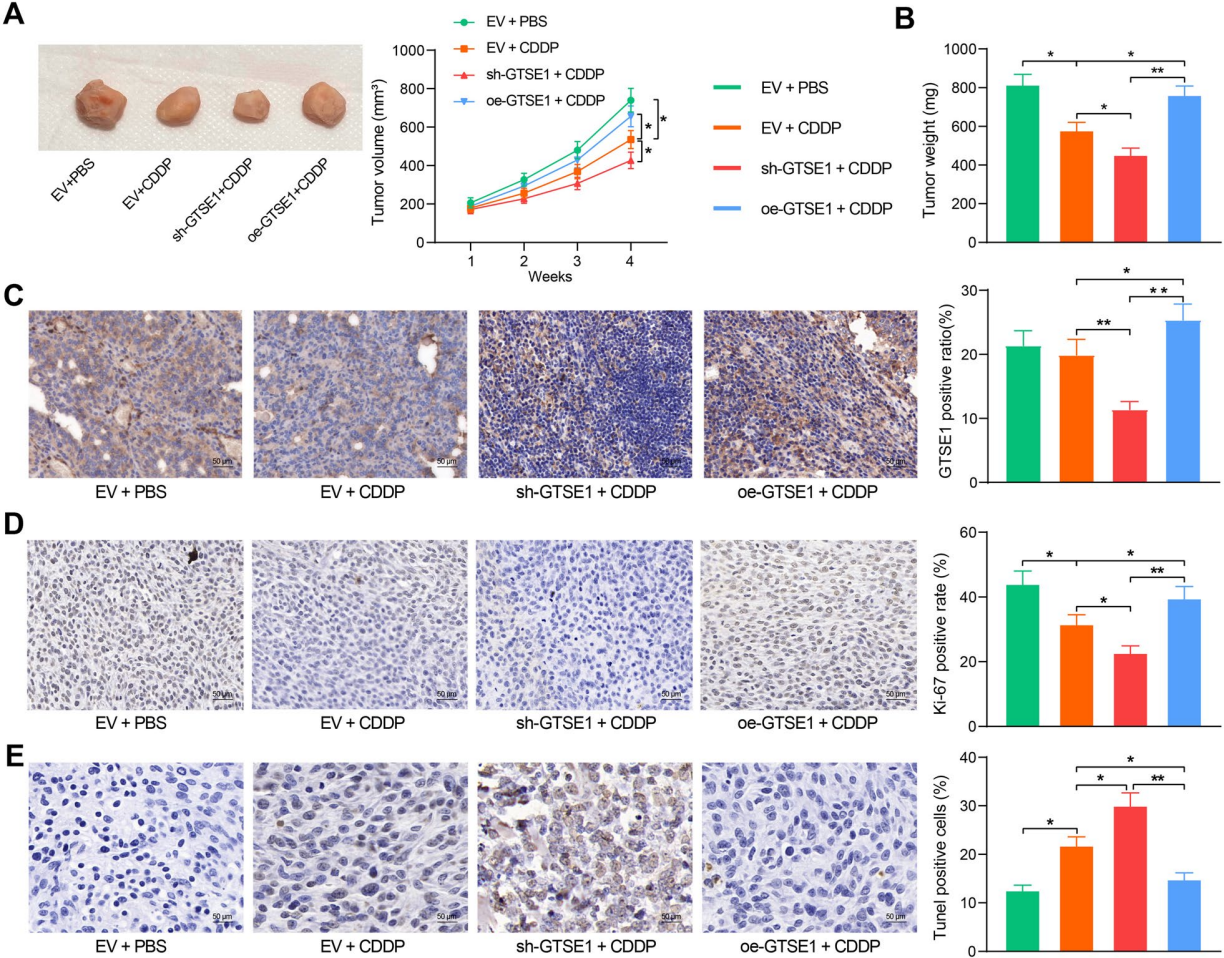
GTSE1 affects tumorigenesis of OS cells in nude mice following CDDP treatment. **A** Growth rate of xenograft tumors in each group of mice (two-way ANOVA); **B** weight of the xenograft tumors in each group of mice (one-way ANOVA); **C**, **D** GTSE1-positive (**C**) and Ki-67-positive (**D**) cells in xenograft tumors examined by IHC staining (one-way ANOVA); **E** portion of apoptotic cells in xenograft tumors determined by TUNEL assay (one-way ANOVA). Data were presented as the mean ± SD from three repetitions. *p < 0.05, **p < 0.01

The function of GTSE1 in CDDP resistance in OS was further examined in vivo. To avoid unnecessary animal death, only MG-63A cells with the highest expression of GTSE1 were used for animal experiments. GTSE1 stably transfected with oe-GTSE1 or sh-GTSE1 were injected into the right flank of nude mice, followed by PBS or CDDP treatment. Compared to PBS treatment, injection of CDDP significantly reduced growth of xenograft tumors in nude mice. Silencing of GTSE1 strengthened, whereas overexpression of GTSE1 blocked the tumor-eliminating function of CDDP in nude mice (Fig. 5A,B). The IHC staining of Ki-67 (a proliferation marker) indicated that silencing of GTSE1 led to a further decline in Ki-67 expression, whereas overexpression of GTSE1 restored the Ki-67 expression in xenograft tumors of mice following CDDP treatment (Fig. 5C,D). The cell apoptosis in the xenograft tumors was examined by TUNEL assay. It was observed that CDDP treatment induced cell apoptosis in the xenograft tumors. Downregulation of GTSE1 further promoted, whereas overexpression of GTSE1 reduced cell apoptosis in the xenograft tumor tissues (Fig. 5E).

## Discussion

The bone microenvironment is a complex biological system and an ideal fertile soil which facilitates the onset and development of both primary and secondary tumors [7]. The survival of patients largely benefited from the advance in chemotherapy, but chemoresistance frequently occurs, leading to treatment failure [23]. In this study, the authors report that the cell cycle-related gene GTSE1 is possibly correlated with activation of the DNA damage repair signaling and reduced treating effect of CDDP on OS.

The expression profiling of GTSE1 in OS has never been mentioned previously, though its high expression has been identified in several malignancies [17, 19–21, 24]. The bioinformatics data indicated that GTSE1 was highly expressed in OS and correlated with unfavorable prognosis of patients. High GTSE1 expression was then detected in clinically collected OS tissues and the procured OS cell lines, preliminarily indicating its involvement in cancer progression. Intriguingly, GTSE1 overexpression has been reported to delay the transition of the G2 to M phase [25]. Likewise, a study by Fen Lin et al*.* suggested that downregulation of GTSE1 increased the portion of breast cancer cells in the S phase, whereas GTSE1 upregulation led to a delay in the M/G2 transition [26]. In agreement with this, in the present study, downregulation of GTSE1 increased the number of MG-63 cells in the S phase while upregulation of GTSE1 increased the number of cells in the G2 phages, suggesting that GTSE1 is crucial for the S/G2 transition in OS cells. A recent study by Xiong et al. demonstrated that high expression of GTSE1 was mainly enriched in the cell cycle, DNA replication, and p53 signaling pathways in prostate cancer [27]. GTSE1 has also been reported to play a tumor-promoting role through triggering cell proliferation [17]. In this paper, the EdU labeling assay indicated that overexpression of GTSE1 augmented the DNA replication ability of OS cells. Collectively, these findings elucidated the oncogenic role or GTSE1 in OS.

DNA repair and apoptosis resistance has been well-established as one of the mechanisms, leading to drug resistance [28]. CDDP represents one of the major DNA damaging drugs in the treatment of OS [11]. It cross-links with nuclear DNA to induce genomic DNA damage, and it can also cytotoxically lead to tumor cell apoptosis via the production of reactive oxygen species [29]. As aforementioned, GTSE1 has been demonstrated to induce DNA damage impair and CDDP resistance by inhibiting the p53 signaling in gastric cancer [18]. Importantly, in our experiments, downregulation of GTSE1 in MG-63 cells reduced the IC_50_ value of CDDP. Namely, it reduced the CDDP-resistance of the cancer cells. As a consequence, the apoptosis rate of cells was increased upon GTSE1 silencing. Overexpression of GTSE1 in 143B cells led to inverse trends. High expression of GTSE1 has been reported to be associated with reduced sensitivity of hepatocellular carcinoma cells to 5-fluorouracil [30]. Likewise, upregulation of GTSE1 by other molecules was correlated with increased CDDP resistance in non-small cell lung cancer (NSCLC) [31]. GTSE1-mediated stabilization of p21 conferred cell resistance to the microtubule cytotoxic paclitaxel [32]. Moreover, in the present study, overexpression of GTSE1 increased the expression of the repair marker DNA-PKcs and the related factors including EXO1, PLK4, and RFC4, and it reduced the expression of DNA damage marker γH2AX in cells. Upon DNA damage, GTSE1 promotes cytoplasmic translocation and degradation of p53 and blocks its pro-apoptotic roles [17]. Quite in agreement with our findings, GTSE1 upregulated γH2AX, whereas suppressed DNA-PKcs in NSCLC to counteract the DNA damage induced by irradiation [24]. Moreover, the function of GTSE1 was further validated in vivo, where knockdown of GTSE1 reduced the volume and weight of xenograft tumors following CDDP treatment, whereas overexpression of GTSE1 blocked the tumor-eliminating role of CDDP.

## Conclusion

In conclusion, this study demonstrates that GTSE1 plays an oncogenic role in OS whose high expression is possibly correlated with cell cycle progression and most importantly, the DNA damage repairs following CDDP treatment. This study may offer novel insights into the treatment of OS that concomitant inhibition of GTSE1 may strengthen the treating effects of CDDP or other DNA damage-related chemo-drugs.

## Data Availability

The data used to support the findings of this study are available from the corresponding author upon request.

## Abbreviations

ANOVA: Analysis of variance
CCK-8: Cell counting kit-8
CDDP: Cisplatin
DMEM: Dulbecco’s modified Eagle’s medium
EdU: 5-Ethynyl-2ʹ-deoxyuridine
EXO1: Exonuclease 1
FBS: Fetal bovine serum
FITC: Fluorescein isothiocyanate
GAPDH: Glyceraldehyde-3-phosphate dehydrogenase
GTSE1: G2 and S phase-expressed-1
IC50: 50% Inhibitory concentration
IgG: Immunoglobulin G
IHC staining: Immunohistochemical staining
mean ± SD: Mean ± standard deviation
NC: Negative control
OD: Optical density
OS: Osteosarcoma
PBS: Phosphate-buffered saline
PCNA: Proliferating cell nuclear antigen
PFA: Paraformaldehyde
PI: Propidium iodide
PLK4: Polo-like kinase 4
RFC4: Replication factor C subunit 4
RT-qPCR: Reverse transcription-quantitative polymerase chain reaction
shRNA: Short hairpin RNA
TUNEL: Terminal deoxynucleotidyl transferase (TdT)-mediated dUTP nick end labeling
